# Network Topology Reconfiguration-Based Blind Equalization over Sensor Network

**DOI:** 10.3390/s24144524

**Published:** 2024-07-12

**Authors:** Chi Sulin, Shimamura Tetsuya

**Affiliations:** 1 Otemon Gakuin University, Osaka City 567-8502, Japan; 2 Department of Science and Engineering, Saitama University, Saitama City 338-8570, Japan; shima@mail.saitama-u.ac.jp

**Keywords:** in-network processing, sensor node, received signal, blind equalizer, mean square error

## Abstract

Distributed in-network processing has garnered much attention due to its capability to estimate the unknown parameter of interest from noisy measurements based on a set of cooperating sensor nodes. In previous studies, distributed in-network processing mainly focused on short-distance communication systems, wherein sensor nodes collect certain parameters of interest within their maximum communication distance. In addition, the estimation of certain parameter vectors of interest from noisy measurements, relying heavily on training signals, is achieved with a non-blind distributed estimation algorithm. However, in some applications, acquiring knowledge of training signals beforehand is difficult. Therefore, it is necessary to perform distributed estimation algorithms for receivers without training signals, a concept known as blind distributed estimation. In this paper, the generalized Sato algorithm is used to design the blind equalizer for the signal estimation. In addition, we consider extending the short-distance communication system to a long-distance communication system for an unmanned aerial vehicle (UAV) cooperating with sensor nodes in the wireless sensor network (WSN). In this scenario, the data signal is transmitted from a UAV to the WSN and is received by sensor nodes. However, the performance of the blind equalizer is susceptible to the transmission channel in long-distance communication systems. Here, we present a network topology reconfiguration approach to address the issue of distributed blind equalization. The objective of the proposed method is to discard the influence of ill-channels on the other sensor nodes by detecting ill-channels and redesigning the sensor node weights. Through computer simulation experiments, we evaluated the performance of the blind equalizer using the average mean square error (MSE) and average symbol error rate (SER). In the results of the computer simulation experiments, the blind equalizer using the proposed method outperformed the conventional methods in terms of prediction accuracy and convergence speed.

## 1. Introduction

With the accelerated development of Internet-of-Things (IoT), wireless sensor networks (WSNs) are gaining importance in various fields, such as military surveillance, precision agriculture, and environmental monitoring. A WSN is a self-organized communication network comprised of numerous small IoT devices known as “sensor nodes”. These sensor nodes are capable of sensing, monitoring, learning, and communicating. They enable flexible cooperative learning and information processing can be performed across a set of spatially distributed sensor nodes, which is known as in-network processing. Depending on the cooperation strategy, in-network processing can be classified into centralized and distributed approaches. In classical centralized in-network processing, a cyclic path and a fusion sensor node are required. In other words, each sensor node is governed by the fusion sensor node [1]. However, centralized in-network processing easily results in the fragility of the dysfuncation of the fusion sensor node. On the other hand, in distributed in-network processing, each sensor node independently collects and shares information with other sensor nodes. This reduces the amount of data communication over the WSN, thereby saving sensor energy and extending the lifetime of the entire network. Due to these merits, compared with centralized in-network processing, distributed in-network processing is considered to be an effective approach for in-network processing [2].

With the conceptual structure of distributed in-network processing, research on distributed adaptive algorithms aims to explore methods for estimating the parameter data of interest from noisy measurements, known as distributed estimation, which has garnered much attention. In previous studies, various approaches were proposed, such as incremental least-mean square (i-LMS) [1,3,4], the incremental affine projection algorithm (i-APA) [5], incremental recursive least square (i-RLS) [3], diffusion LMS (d-LMS) [6,7,8], and diffusion RLS (d-RLS) [9,10]. However, most of distributed estimation approaches, such as [3,6,8], and [10], primarily focus on the study of unknown parameter estimation assuming knowledge of the training signal or the desired signals in advance. However, in most practical applications, it may be difficult to obtain the training signal in advance. Furthermore, even if the training signal is accessible in some applications, valuable channel capacity may be sacrificed [11,12,13].

Therefore, to compensate of the drawbacks of distributed estimation, where the unknown parameter estimation relies on the availability of training signals, distributed blind estimation was developed for transmitted data signal estimation without training signals or desired signals. Analyzing the performance of blind estimation algorithms is theoretically challenging due to the minimization of nonlinear cost functions during adaptation, especially when considering the effect of distributed cooperation among sensor nodes in WSNs. Communication among sensor nodes has usually been constrained to a single hop with a Hamiltonian cycle, rendering it susceptible to the failures of sensor nodes [14]. Indirect distributed estimation algorithms were derived in [15]. Nevertheless, the computational complexity of the algorithm in [15] is exceedingly high, leading to a time-consuming implementation. In the literature, in order to estimate the transmitted data signal, various approaches for blind adaptive algorithms have been proposed for equalizing the transmission channel [16,17,18]. In [2], to enhance the performance of the blind distributed estimation algorithm, a combination approach between a WSN and the estimation algorithm, including the combine-then-adaptive generalized Sato algorithm (CTA-GSA) and the adaptive-then-combine GSA (ATC-GSA), was proposed. In [19], the optimal channel output is selected in the WSN to improve the performance of distributed blind equalization. Additionally, in [20], the weight of each sensor node employed is adjusted to alleviate the impact between inter-connected sensor nodes, thereby enhancing the performance of the blind equalization. However, some limitations exist, such as in [2], where a good performance was achieved only under common channel and noise conditions, and in [19], where knowledge of all sensor node information is required at the receiver. In [21,22], the performance of the blind equalization under ill-channel conditions was improved by redesigning the weight of each sensor node. In [23], in order to allow the blind equalizer to adapt to the varying channel conditions, the weight of each sensor node was assigned based on the estimation error of the blind equalization. In [24], the authors proposed a method to find the optimal local sensor network to improve the performance of distributed blind equalization.

In this paper, the challenge of distributed blind estimation over a WSN is considered. We employ the diffusion cooperation rule, where each sensor node cooperates only with its neighbors, to reduce the restriction on the incremental cooperation rule by the network topology [14]. That is, the number of the entire WSN cannot be known by any single sensor node. Additionally, to enhance transmission efficiency and reduce computation, the transmitted data signal is estimated based only on the received signal at each sensor node without prior knowledge of training signals or desired signals. We propose a network topology reconfiguration approach to improve the existing network topology based on the received signals at each sensor node in the WSN. We consider discarding the detrimental impact of ill-channel conditions on the other sensor nodes to improve the performance of distributed blind estimation. This is a key tenet since the distributed blind estimation algorithm is insensitive to transmission channel conditions, especially in cases of ill-channel conditions. The performance of distributed blind estimation using the proposed approach, which poses significant challenges in the context of blind signal processing, was verified using both the average mean square error (average MSE) and average symbol error rate (average SER).

The organization of this paper is as follows: Section 2 formulates the system model and the problem of distributed in-network processing. Section 3 presents details of both distributed blind equalization and the proposed method. The computer simulation is presented in Section 4. Finally, this paper is concluded in Section 5.

## 2. System Model and Problem Formulation

**Figure 1:**
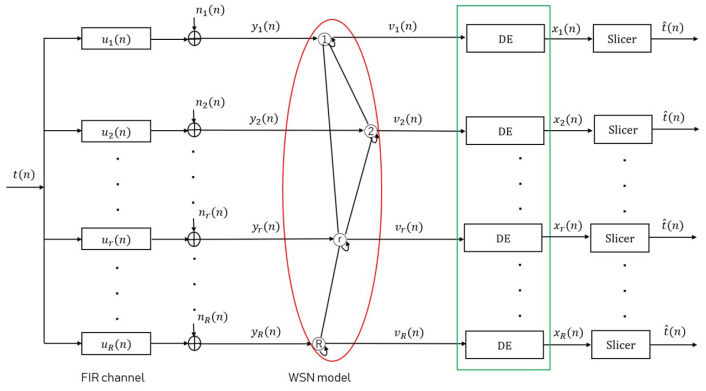
System model. “DE” denotes distributed blind equalizer.

In this paper, let us consider a WSN that consists of *R* sensor nodes. These sensor nodes are distributed spatially within a certain network topology. An undirected graph is utilized to describe the network topological structure, where, if two sensor nodes are connected by a line without arrows, then two sensor nodes can communicate and share information with each other. Additionally, each sensor node is assumed to be connected to itself. With reference to Figure 1, all sensor nodes in the WSN are interested in a common data signal t(n). As this data signal t(n) passes through finite impulse response (FIR) channel $u_{r}\left(n\right)$ and is collected at each sensor node *r*, it results in a channel output signal $y_{r}\left(n\right)$, depicted as

(1) $$\begin{array}{cc} y_{r}\left(n\right) & =\sum_{i=0}^{I-1}u_{r}\left(i\right)t\left(n-i\right)+n_{r}\left(n\right)\\ \; & =u_{r}\left(n\right)⊛t\left(n\right)+n_{r}\left(n\right) \end{array}$$

where the additive measurement noise is described as $n_{r}\left(n\right)$ at sensor node *r*, which follows a complex circular Gaussian distribution $C(0,\sigma_{r}^{2})$. Otherwise, the data signal t(n) is represented using a typical modulation signal with a constant envelope, such as 4 Quadrature Amplitude Modulation (4QAM).

**Figure 2:**
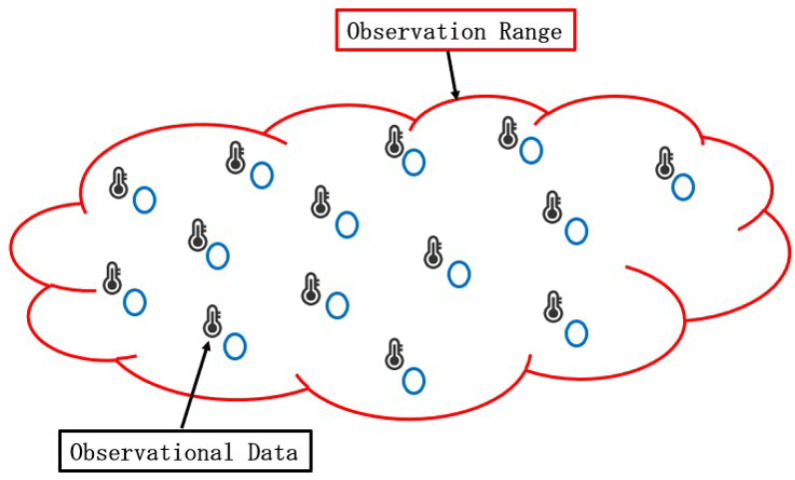
Two-dimensional network model for distributed in-network processing.

**Figure 3:**
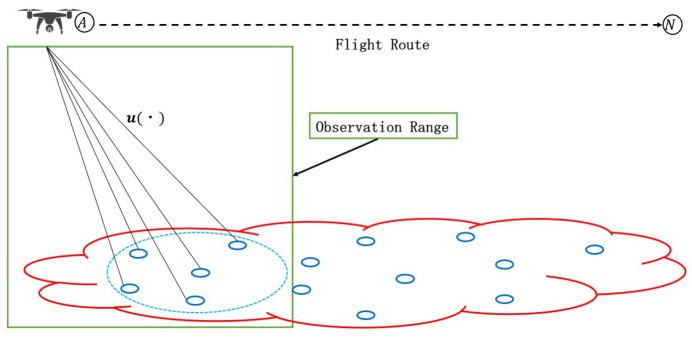
Three-dimensional network model for distributed in-network processing.

In conventional studies of WSN models, each sensor node can only measure information of interest within its maximum communication distance, as illustrated in Figure 2. Information beyond this observation range cannot be collected by the WSN. This kind of data transmission model is commonly referred to as the two-dimensional (2D) distributed network model. In contrast to the 2D distributed network model, in this paper, we consider a network model for distributed signal processing that can be applied in a 3D system model, as depicted in Figure 3. In other words, the main differences between 2D and 3D models are the transmission channel and the estimation object. For a 2D model, it is primarily used for the estimation of unknown parameters collected by each sensor node, without considering the transmission channel. However, the 3D model focuses on estimating the transmitted data signal received by each sensor node, and the transmission channel must be taken into account. In addition, in computation simulations, the network topology is represented by two-dimensional axes for both 2D and 3D models. In this model, low-cost sensor nodes are deployed on the ground to receive the transmitted data signal, while information of interest is collected by an unmanned aerial vehicle (UAV). Recently, UAVs, also known as drones, have seen tremendous advancements in supporting technologies [25,26,27]. UAVs offer high mobility and plug-and-play features, making it easy to directly collect information around a sensor node, thereby saving the energy that would otherwise be consumed by sensor nodes in gathering this information [27,28]. In addition, the UAV can also communicate directly with various sensor nodes to hand over the information for transmission [27,28], thus avoiding the high energy consumption associated with direct transmission by the UAV itself. The UAV method enhances the reliability of data transmission. The collected information of interest is rapidly transmitted as the common data signal by a UAV flying over a part of the WSN, and the broadcasting information lasts for a very short period of time [2,29,30]. During this short period of time, the common transmitted data signal is received by sensor nodes within a segment of the entire WSN area. With reference to Figure 3, we assume that a UAV starts flying from location Ⓐ. Thus, the UAV can only communicate with a subset of sensor nodes within the entire WSN, marked by the blue circle. During this time, to communicate with sensor nodes, the UAV remains stationed at “Ⓐ”. Subsequently, the UAV flies from location “Ⓐ” to “Ⓝ”, following the fight route depicted as a dotted line with an arrow in Figure 3. Under this assumption, it is reasonable to assume that the transmission channel u(n) applied to the data signal differs at each sensor node. Otherwise, sensor nodes might easily be damaged, since they are usually deployed in harsh environments. It is crucial to note that, in some scenarios, if only one sensor node is arranged at the receiver, the lost information cannot be recovered. Therefore, the adoption of a distributed sensor network is a good candidate, which is widely used in wireless cooperative communication [29,30].

Considering the system model assumed in Figure 3, in this paper, a distributed sensor network is used to collaboratively estimate the common data signal t(n) based only on the received signal at each sensor node, $y_{r}\left(n\right)$, without knowing the information of the transmission channel or noise. However, the performance of the distributed estimation algorithm can easily be affected by the transmission channel, especially under ill-channel conditions. A network topology reconfiguration approach is proposed to address this challenge, thereby improving the existing network topology and reconfiguring the weight for each sensor node. Then, an optimal estimate for the common data signal t(n) is obtained using a suitable slicer ζ(·) of the distributed estimation algorithm. It is also noted that, here, no noise or distortion occurs in the WSN.

## 3. Network Topology Reconfiguration Approach for Distributed Blind Estimation

### 3.1. Distributed Blind Equalization

In this section, our objective is to develop a distributed blind adaptive algorithm for designing a blind equalizer and estimating the transmitted data signal based solely on the received signal in the WSN. In this paper, we employ the GSA [31] for designing the blind equalizer.

Based on the system model outlined in Section 2, each sensor node corresponds to a distinct transmission channel. Consequently, unlike the traditional centralized model, the optimization problem cannot be derived at a single sensor node. In the distributed system model, the optimization problem varies for each node. However, in the diffusion model, this problem cannot be directly solved due to the unavailability of information from all sensor nodes at a single sensor node.

In a WSN, each sensor node is only allowed to access the information of its neighbors. The neighbors of the sensor node *r* are described as $N_{r}$, representing the sensor node *r* and its directly connected sensor node, including itself. The weight of the neighbors of the sensor node *r* and the FIR channel $y_{r}\left(n\right)$ are used to aggregate the sensor output $v_{r}\left(n\right)$, which is defined as

(2) $$\begin{array}{c} v_{r}\left(n\right)=\sum_{m\in N_{r}}c_{m,r}y_{m}\left(n\right) \end{array}$$

where the coefficients $c_{m,r}$ are non-negative elements in the R×R combination weight matrix **C**. These coefficients $c_{m,r}$ shows that the sensor node *m* shares its own data with the sensor node *r*, where the sensor node *m* is a neighbor of the sensor node *r*. In this paper, the Euclidean distance and the maximum communication distance are used to define whether two sensor nodes are neighbors. If the Euclidean distance between two sensor nodes is less than or equal to the maximum communication distance, then they are considered connected and share information with each other, i.e., neighbors. Otherwise, they are not connected. Additionally, each sensor node in a WSN is limited to single-hop communication exclusively with its neighbors. It is important to note that the total number of sensor nodes *R* is assumed to be unknown to each individual sensor node. The aggregated sensor output $v_{r}\left(n\right)$ serves as the equalizer input signal for estimating the transmitted data signal t(n).

Therefore, in this paper, the approximated cost function is considered at sensor node *r*, which is described as

(3) $$\begin{array}{cc} \Gamma_{r}\left(n\right) & =E[|\lambda \mathrm{csgn}\left(x_{r}\left(n\right)\right)-x_{r}\left(n\right){|}^{2}] \end{array}$$

where the symbol “*E*” denotes the statistical expectation, and the parameter “λ” is a positive constant used to set the gain of the blind equalizer, depending only on the transmitted data signal t(n), which is given by

(4) $$\begin{array}{c} \lambda =\frac{E[|t_{r}\left(n\right){|}^{2}]}{E[|t_{r}\left(n\right)|]}=\frac{E[|t_{i}\left(n\right){|}^{2}]}{E[|t_{i}\left(n\right)|]} \end{array}$$

where $t_{r}\left(n\right)$ and $t_{i}\left(n\right)$ are the real and imaginary parts of the transmitted data signal t(n), respectively. In (3), “csgn” denotes a complex “sign” function for the complex data symbol, which is described as

(5) $$\begin{array}{c} \mathrm{csgn}\left(x_{r}\left(n\right)\right)=\mathrm{sign}\left(x_{rr}\left(n\right)\right)+j\mathrm{sign}\left(x_{ri}\left(n\right)\right) \end{array}$$

where $x_{rr}\left(n\right)$ and $x_{ri}\left(n\right)$ are the real and imaginary parts of the equalizer output $x_{r}\left(n\right)$, respectively. $x_{r}\left(n\right)$ is defined as

(6) $$\begin{array}{c} x_{r}\left(n\right)=v_{r}^{T}\left(n\right)z_{r}\left(n\right) \end{array}$$

where $v_{r}\left(n\right)$ denotes the input vector of the blind equalizer at the sensor node *r*, which is defined as $v_{r}\left(n\right)=\left[v_{r}\left(n\right),v_{r}\left(n-1\right),\ldots ,v_{r}\left(n-N+1\right)\right]$, and $z_{r}\left(n\right)$ denotes the tap coefficient vector of the blind equalizer, which is given by $z_{r}\left(n\right)=\left[z_{r1}\left(n\right),z_{r2}\left(n\right),z_{r3}\left(n\right),\ldots ,z_{rN}\left(n\right)\right]$, where “*N*” indicates the length of the blind equalizer and the notation “*T*” denotes transpose.

Therefore, the estimation error sequence of the blind equalizer at the sensor node *r* is described as

(7) $$\begin{array}{c} \varrho_{r}\left(n\right)=\lambda \mathrm{csgn}\left(x_{r}\left(n\right)\right)-x_{r}\left(n\right) \end{array}$$

and the tap coefficients of the blind equalizer are given by

(8) $$\begin{array}{c} z_{r}\left(n+1\right)=z_{r}\left(n\right)+\mu \varrho_{r}\left(n\right)v_{r}^{*}\left(n\right) \end{array}$$

where the parameter μ is described as the step size of the GSA and the notation “*” is the complex conjugate for the sensor node *r*.

### 3.2. Proposed Approach

Distributed blind equalization is a valuable technique, which is usually used to equalize the channel and estimate the transmitted data signal without prior knowledge of the transmission channel. This method focuses on equalizing the channel directly rather than estimating it, thereby addressing issues such as short network lifetime and the consumption of bandwidth and energy, which are common in distributed estimation algorithms. However, in the blind equalization for estimating the transmitted data signal, each sensor node not only utilizes its own data but also incorporates data from its neighbors. Thus, the performance of each sensor node might be affected by its neighbors, resulting in the performance of blind equalization being affected by the received signal in a WSN. To address this problem and improve the performance of the distributed blind equalizer, a network topology reconfiguration approach is proposed. The proposed approach aims to mitigate the affect of ill-channel conditions.

In conventional methods, various methods have been proposed to improve the performance of the blind equalizer by assigning weights to each sensor node, including the Metropolis [32,33], the Laplacian [32], the Uniform (average) [34,35], the Relative-degree [10], and the Maximum degree [36] combination weight rules. However, the weight of each sensor node is assigned based only on the degree of each sensor node in [10,32,33,34,35,36]. In some situations, such as in the presence of an ill-channel condition, blind equalization may not achieve optimal performance using these combination weight rules [10,32,33,34,35,36]. To overcome this disadvantage, the Metropolis-Hasting [33,37] and the Relative-degree variance [8] combination weight rules have been proposed. However, the noise information should be known or an accurate estimation in advance is needed, as in [8]. In practice, for efficiency reasons, it may be physically infeasible to obtain the noise variance in most practical applications. In [38], the information of noise may be offered based on signal-to-noise ratio (SNR) estimation, but it is achieved when the signal power surpasses the noise power. In [39], SNR estimation algorithms depend on specific conditions, such as knowledge of signal boundaries or stationary noise backgrounds. Furthermore, SNR estimation for practical implementation poses challenges, including the need for intricate calculations [38] and the requirement for a small step size [37] when applying the Metropolis-Hasting combination weight rule for distributed estimation algorithms, making it less feasible for real-world applications.

In this paper, we develop a network topology reconfiguration method that aims to mitigate the impact of ill-channels on the distributed blind equalizer. In order to analyze the transmission channel conditions, the condition number of the received vector is utilized at each sensor. However, analyzing the transmission channel condition solely based on individual signals can be challenging. Hence, we developed the concept of the local sensor network (LSN) to analyze the corresponding channel conditions. The *r*-th LSN is defined as the set of sensor nodes comprising the sensor node *r* and its neighbors. The proposed method involves the detection of the ill-channel and the re-assignment of weights to each sensor node in each LSN. For ill-channel detection, we analyze the channel condition using the condition number and remove the ill-channels from each LSN. Subsequently, the weight of the rest of the sensor nodes can be assigned based on the power of the received signal at each LSN. This is to ensure that the received signal, which is transmitted via well-channels, can be better utilized for signal estimation, thereby improving the prediction accuracy of the blind equalizer and reducing the effect of the ill-channel on the other sensor nodes. The criteria for ill-channel detection and weight assignment, outlined in this section, ensure a more effective reduction of this impact on the blind equalization process.

#### 3.2.1. Ill-Channel Detection

Based on the assumptions of the system model, it is acknowledged that the conditions of the transmission channel vary with the movement of a UAV over time. In [40], an approach for detecting impulse noise (IN) was proposed to improve the performance of the distributed LMS algorithm. The authors demonstrated that the performance of the distributed LMS can be improved by discarding the IN-contaminated data, essentially creating an IN-free environment at each sensor node. However, in [40], this approach requires the availability of the desired signal for data estimation and is most effective in short-distance communication systems, where the estimated data signals can be collected within the maximum communication distance of the WSN. In contrast, in this paper, the received signal at each sensor node is used to estimate the channel condition without relying on the desired signal, making it suitable for long-distance communication system. In order to calculate the channel condition, we developed the condition number, which is a very important tenet for measuring the condition of the transmission channel based on the auto-correlation matrix of the received signal at each sensor node. The condition number reflects the influence of the channel on the transmitted signal. If the condition number of channel is small, the transmitted data signal has a low correlation through the transmission channel. Otherwise, the transmitted data signal has strong correlation through the transmission channel when the condition number of the received signal is large. In other words, a small condition number means that the received signal has little distortion, and a large condition number means that the received signal has a lot of distortion.

In the *r*-th LSN, the channel condition of each sensor node is calculated from the correlation matrix, $Q_{r}$, at the sensor node *r*. The matrix $Q_{r}$ is a K×K Toeplitz matrix, which is described as

(9) $$Q_{m}=\left[\begin{array}{cccc} Q_{m}\left(0\right) & Q_{m}\left(1\right) & \cdots & Q_{m}\left(K-1\right)\\ Q_{m}\left(1\right) & Q_{m}\left(0\right) & \cdots & \vdots \\ \vdots & \vdots & \ddots & \vdots \\ Q_{m}\left(K-1\right) & Q_{m}\left(K-2\right) & \cdots & Q_{m}\left(0\right) \end{array}\right],\;\;\;\;\;m\in N_{r}$$

where $Q_{m}\left(n\right)$ is calculated based on the channel output signal $y_{m}\left(n\right)$ at the sensor node *m*, where *m* is the neighbors of the sensor node *r*, and *K* denotes the number of data samples. The condition number of each sensor node in the *r*-th LSN is calculated as

(10) $$\begin{array}{c} G_{m}=\frac{\lambda_{m}^{max}}{\lambda_{m}^{min}},\;\;\;\;\;m\in N_{r} \end{array}$$

where $\lambda_{m}^{max}$ and $\lambda_{m}^{min}$ represent the maximum and minimum eigenvalues of $Q_{m}$, respectively. The corresponding channel condition is determined by the magnitude of $G_{m}$. While a more accurate evaluation of $G_{m}$ can be obtained with larger values of *K*, but this also leads to an increased computation time [41,42]. Therefore, a certain size for *K* was chosen in the proposed method to balance accuracy and computational efficiency.

**Figure 4:**
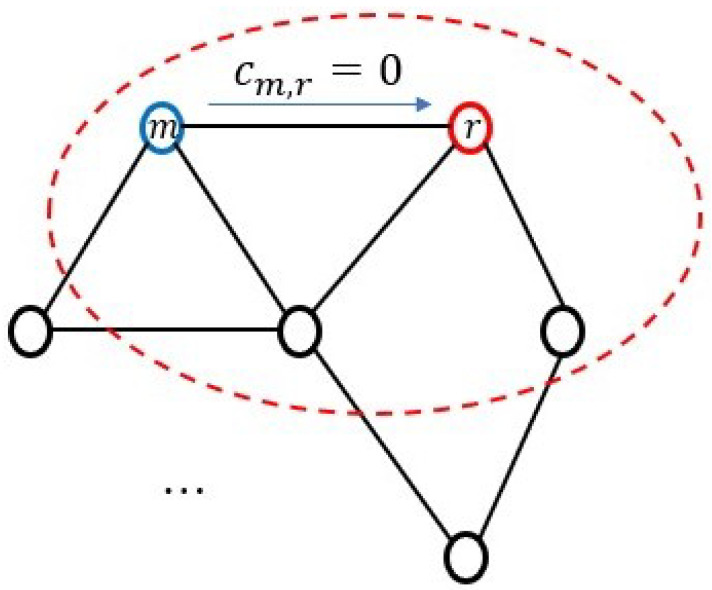
Ill-channel detected in the neighbor of the *r*-th LSN.

As we mentioned in Section 2, it is a challenge to obtain the knowledge of the channel condition when relying on a single sensor node only. In this paper, the identification of sensor nodes corresponding to ill-conditioned channels in the *r*-th LSN is inferred by comparing the condition numbers of each sensor node within this LSN. In the *r*-th LSN, the ill-channel detection approach has the following three steps:

1. The maximum condition number in the *r*-th LSN is calculated as
  (11) $$\begin{array}{c} G_{m}^{max}=\mathrm{Max}\left[G_{r}\right] \end{array}$$
  where the function Max[·] denotes the maximum function used to find the maximum value of all sensor nodes in the *r*-th LSN from the condition number vector $G_{r}$;
2. The minimum condition number in the *r*-th LSN is calculated as
  (12) $$\begin{array}{c} G_{m}^{min}=\mathrm{Min}\left[G_{r}\right] \end{array}$$
  where the function Min[·] denotes the minimum function used to find the minimum value of the condition number in the *r*-th LSN;
3. The ill-channel is detected for the current LSN by comparing the maximum and minimum condition numbers.
  (13) $$\begin{array}{c} \mathrm{Ic}=\mathrm{Com}\left[\alpha G_{m}^{max},G_{m}^{min}\right],\;\;\;\;\;m\in N_{r} \end{array}$$
  where the function Com[·] means that the comparison between the maximum and minimum values, α is a positive constant, which satisfies 0<α<1. If the value of $\alpha G_{m}^{max}$ is greater than $G_{m}^{min}$, then we consider those sensor nodes corresponding to ill-channels, where the condition numbers of sensor nodes are larger than $\alpha G_{m}^{max}$. Consequently, the weights of those sensor nodes are set as 0. On the other hand, if the value of $\alpha G_{m}^{max}$ is less than or equal to $G_{m}^{min}$, then the sensor node with the value $\alpha G_{m}^{max}$ is defined as the ill-channel. For example, in Figure 4, if the neighbor *m* of the sensor node *r* is detected to correspond to an ill-channel in the *r*-th LSN, the weight of the sensor node *m* is set as 0, ($c_{m,r}=0$). Moreover, a value of α closer to 1 makes the system more sensitive to ill-channels. However, in each LSN, if α=1, only the best channel can be used for signal estimation, leading to increased power consumption of the sensor node. In order to avoid this situation, an appropriate α needs to be chosen.

#### 3.2.2. Weight Assignment

In the *r*-th LSN, the weights are assigned to the rest of the sensor nodes based on the power of the received signal. Several conditions must be satisfied for the proposed combination weight rule. Firstly, in the *r*-th LSN, all coefficients of the sensor node *r* and its neighbors are required to be both non-negative and non-zero, which are described as

(14) $$\begin{array}{cc} c_{m,r} & >0,\;\;\;m\in N_{r},\;\;\mathrm{and}\;\;\alpha G_{m}^{max}<G_{m}^{min}\\ c_{m,r} & =0,\;\;\;m\neq N_{r} \end{array}$$

When the received signal at each sensor node is used in the *r*-th LSN for communication, then this condition must be satisfied. Therefore, the proposed combination weight rule was designed based on this condition.

When the estimation error of distributed blind equalization becomes small, then an accurate prediction of the transmitted data signal can be provided by distributed blind equalization [43]. Therefore, in order to further improve the influence of the sensor node that corresponds to the noise-free channel on the other sensor nodes, weights are assigned to each sensor node based on the signal power of the received signal. In the *r*-th LSN, the proportion of the received signal power of each sensor node is described as

(15) $$\begin{array}{c} P_{m,r}=\frac{P_{m}}{\sum_{i\in N_{r}}P_{i}},\;\;\;m\in N_{r} \end{array}$$

where $P_{m}$ is the received signal power of the sensor node *m*, and $\sum_{i\in N_{r}}P_{i}$ denotes the sum of the power of all sensor nodes in the *r*-th LSN. The signal power of the common data signal transmitted in a noisy channel is larger than this signal transmitted under a noise-free channel. Therefore, in (15), a large value of $P_{m}$ can be obtained when the data signal is transmitted in a noisy channel condition. However, a smaller weight of this sensor node will be assigned; thus, the inverse of $P_{m,r}$ is taken as $\frac{\varepsilon }{P_{m,r}}$, where ε is a positive constant that satisfies

(16) $$\begin{array}{c} \sum_{m\in N_{r}}\frac{\varepsilon }{P_{m,r}}=1 \end{array}$$

where the sum of all weights assigned to sensor nodes is required to be 1 in the *r*-th LSN. The weight designed for each sensor node is defined as

(17) $$c_{m,r}=\left\{\begin{array}{cc} \frac{\varepsilon }{P_{m,r}}, & \mathrm{if}\;m\in N_{r},\;\;\mathrm{but}\;\;\alpha G_{m}^{max}<G_{m}^{min}\\ 0, & \mathrm{otherwise} \end{array}\right.$$

where $\left\{c_{m,r}\right\}$ is a coefficient in the R×R combination weight matrix C. The matrix C is satisfied as

(18) $$C^{T}1=1,\;\;\;\mathrm{and}\;\;\;c_{m,r}=0,\;\;\mathrm{if}\;\;m\neq N_{r}$$

where **C** is a left-stochastic matrix, in which the entries in each of its columns add up to one.

We implemented distributed blind equalization based on the above proposed combination weight rule. In that case, compared with the CTA-GSA, the amount of computation (complex multiplications and complex additions) was reduced for each update of the tap coefficients in blind equalization in each local LSN, which is shown in Table 1.

In this section, one of the most important properties of the proposed method is highlighted: the accurate prediction of distributed blind equalization is improved by discarding the ill-channel condition data from the received signal and increasing the influence of the well-channel on the other sensor nodes. In other words, well-channel environments can be established for signal estimation in blind equalization. In this manner, both an insensitivity to ill-channels and good estimation performance in blind equalization can be accomplished.

## 4. Simulation Experiments

We assumed that the UAV is flying based on the model in Figure 3 and transmitting the data signal [2,21,22]. We did not assume a specific UAV. However, a general but realistic situation was considered. In order to demonstrate the effectiveness of the proposed approach for blind equalization, we present two cases of network topology.

**Figure 5:**
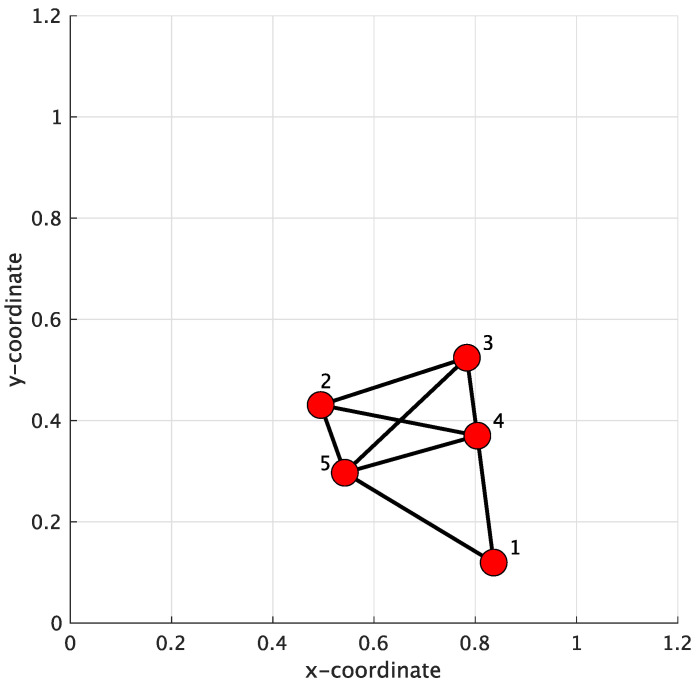
Initial network topology of 5 sensor nodes.

Case 1:

In the simulation of Case 1, a WSN consisting of 5 sensor nodes arbitrarily distributed over a 1.2 m × 1.2 m square area was established, which is shown in Figure 5. The maximum communication distance was set to 0.35 m. The initial network topology defines which sensor nodes can be connected to other nodes in the WSN for conventional methods. However, in the proposed method, the network topology was improved based on the initial network topology. Moreover, in conventional methods, such as the Metropolis [32,33], the Laplacian [32], the Uniform (average) [34,35], the Relative-degree [10], and the Maximum degree [36] combination weight rules, the network topology only changes when some sensor nodes are damaged. In [10,32,33,34,35,36], the Metropolis combination weight rule exhibited better performance than the others [10,15]. Thus, in this paper, the Metropolis combination weight rule was used to compare the conventional methods with the proposed approach.

In this simulation, the raised-cosine channel was used to establish the transmission channel [43] for each sensor node, which is described as

(19) $$u_{r}\left(n\right)=\left\{\begin{array}{cc} \frac{1}{2}\left[1+\cos\left(\frac{2\pi }{\theta }\left(n-2\right)\right)\right], & n=1,2,3\\ 0, & \mathrm{otherwise} \end{array}\right.$$

where the parameter θ controls the amplitude produced by the transmission channel. Increasing the θ parameter increases the distortion of the transmission channel, thus making the channel condition ill [43]. In this case, the parameter θ for each sensor node was randomly set to be within (3.0,3.2,3.4,3.6,3.8). The SNR was used to measure the additive measurement noise n(n) for each sensor node, which was measured as

(20) $$\begin{array}{c} \mathrm{SNR}=\frac{\mathrm{the}\;\mathrm{power}\;\mathrm{of}\;u_{r}\left(n\right)⊛t\left(n\right)}{\mathrm{the}\;\mathrm{power}\;\mathrm{of}\;n_{k}\left(n\right)} \end{array}$$

The noise variance was calculated to maintain SNR=20 dB when the parameter θ=3.4 was used for each channel. For the common transmitted data signal, 4QAM sequences were used.

In this paper, the non-cooperative GSA (Nc-GSA) and the state-of-art method, CTA-GSA, were used as to compare the conventional methods with the proposed method. In the Nc-GSA, the tap coefficient $z_{r}\left(n\right)$ of the *r*-th blind equalization is updated directly from the received signal, which is the channel output signal $y_{r}\left(n\right)$, without incorporating the weight of the sensor node. In contrast, in the CTA-GSA, the tap coefficient of blind equalization is combined with the weight of each sensor node first, and the estimate of $z_{r}\left(n\right)$ at the sensor node *r* can be obtained. The Metropolis rule was used to design the weights for the conventional methods, and the results are shown in Table 2. The Metropolis combination matrix, as shown in Table 2, is a special symmetric matrix in which the entries in each row and column equal one. In Table 2, each row represents each sensor node, and the corresponding columns represent the neighbors of that sensor node, e.g., the entry in the 1-st row and 1-st column indicates that sensor nodes 1, 4, and 5 are connected to sensor node 1, and these sensor nodes (1, 4, and 5) compose the 1-st LSN. The average MSE was used to evaluate the performance (i.e., the convergence behaviors) of blind equalization. And, the average SER was used to evaluate the accurate prediction of blind equalization. In order to maintain the computational efficiency of blind equalization, a constant step-size and equalizer length were set as μ=0.0012 and N=12, respectively.

**Figure 6:**
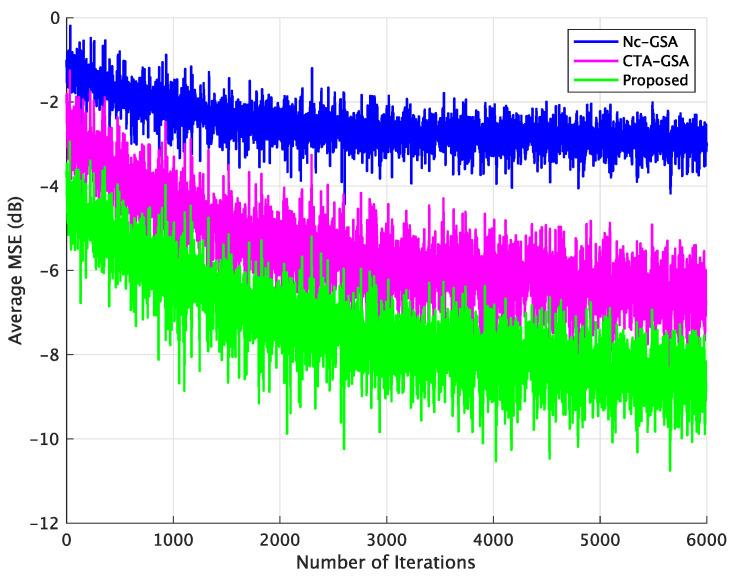
Average MSE comparison between the Nc-GSA, CTA-GSA, and the proposed methods for constant channel condition of Case 1.

Figure 6 displays the results of the average MSE for the Nc-GSA, CTA-GSA, and the proposed methods, with 6000 data samples being used for the evaluation. In Figure 6, a smaller average MSE was achieved with the proposed method. For the conventional methods (Nc-GSA and CTA-GSA), 6000 data samples were utilized to obtain the convergence of blind equalization for each sensor node in each LSN. However, for the proposed method, the $G_{r}$ was utilized to discard ill-channels. We consider that 1000 data samples were sufficient, and the elements of $Q_{r}$ approximately consist of $E[Q_{m}\left(k\right)]$, where k=1,2,…,K and $m\in N_{r}$. The sensor nodes corresponding to ill-channel conditions were not used for the progress of transmitted data signal estimation in the proposed method.

**Figure 7:**
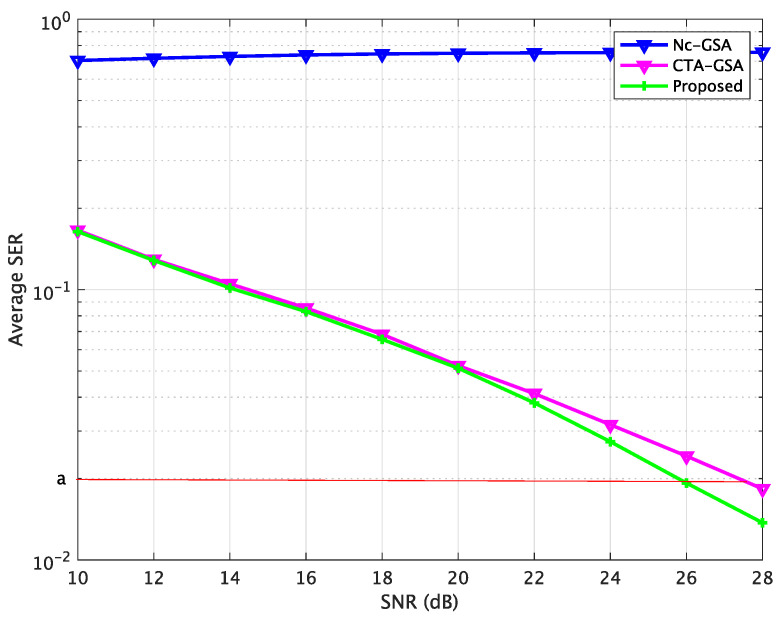
Average SER comparison between the Nc-GSA, CTA-GSA, and the proposed methods for constant channel condition of Case 1.

Figure 7 shows the average SER performance characteristics of the Nc-GSA, CTA-GSA, and the proposed methods, where the data samples were set to $10^{5}$ for each sensor node, as computed by the SER after convergence (6000 iterations). In Figure 7, the SER is relatively larger without cooperation. Blind equalization using the proposed method resulted in a significantly reduced SER, approximately 1.8 *dB* less than the CTA-GSA at the same level, which is shown in Figure 7. The average SER using different approaches is summarized in Table 3. Thus, we also found that blind equalization using the proposed method could be improved compared with the Nc-GSA and CTA-GSA. Therefore, a more accurate estimation of blind equalization could be achieved with the proposed approach.

**Figure 8:**
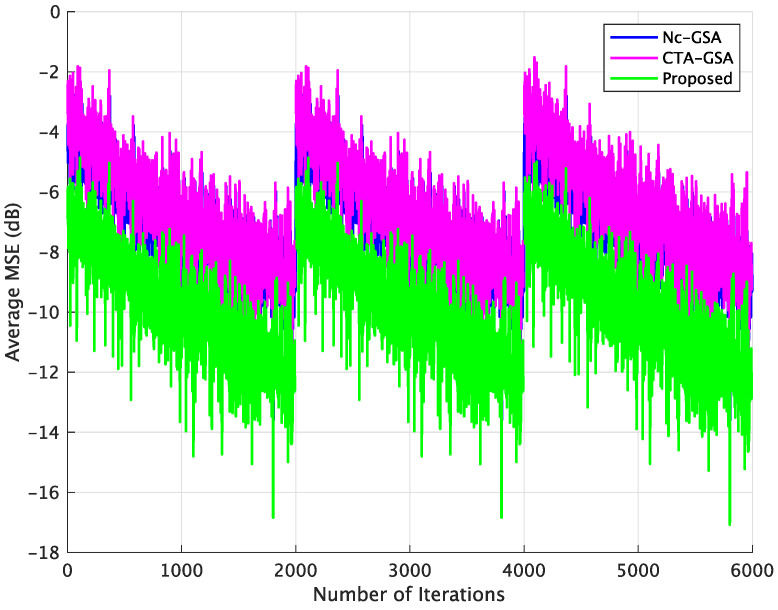
Average MSE comparison between the Nc-GSA, CTA-GSA, and the proposed methods for varying channel condition of Case 1.

In order to evaluate the performance of blind equalization using the proposed method in practical applications, varying channels were also utilized in this case. The parameter θ of the transmission channel for each sensor node varied with the 2000 data samples and was randomly set to be within (3.0,3.2,3.4,3.6,3.8). Figure 8 displays the result of the average MSE comparison between the Nc-GSA, CTA-GSA, and the proposed methods. In Figure 8, we also see that blind equalization using the proposed method achieved a lower MSE.

**Figure 9:**
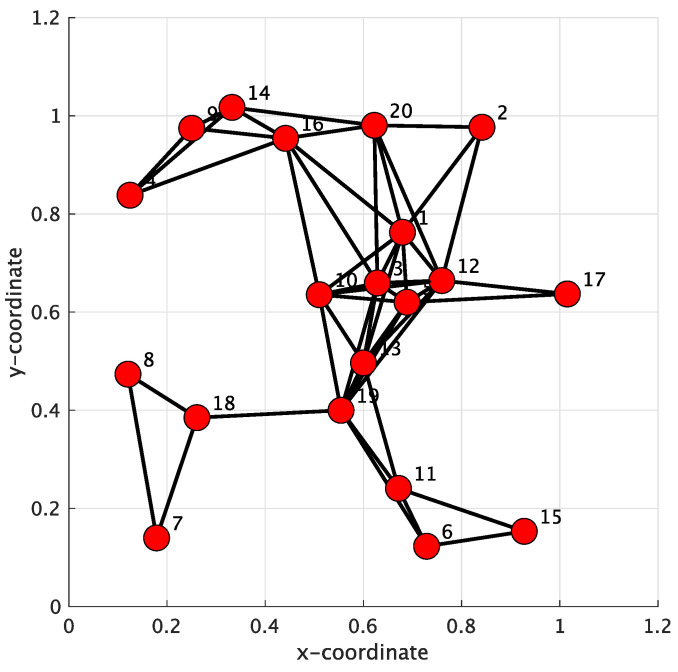
Initial network topology of 20 sensor nodes.

Case 2:

In this case, a WSN consisting of 20 sensor nodes was established over a 1.2 m × 1.2 m square area, which is shown in Figure 9. The Metropolis rule was also used for the conventional method. The combination weight matrix is too large and complex to be shown here. The common settings of blind equalization were also applied in this case. For the setting of the transmission channel, the parameter θ of the transmission channel was randomly set to be from 3.0 to 3.8 for each sensor node.

**Figure 10:**
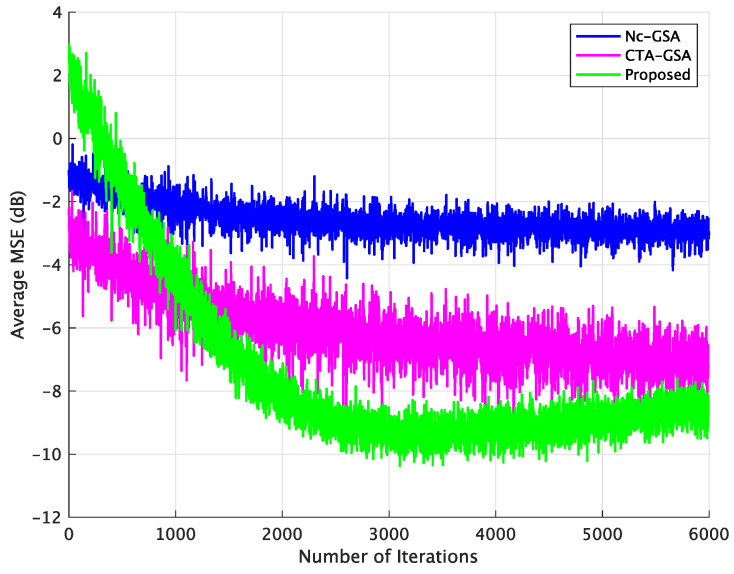
Average MSE comparison between the Nc-GSA, CTA-GSA, and the proposed methods for constant channel condition of Case 2.

**Figure 11:**
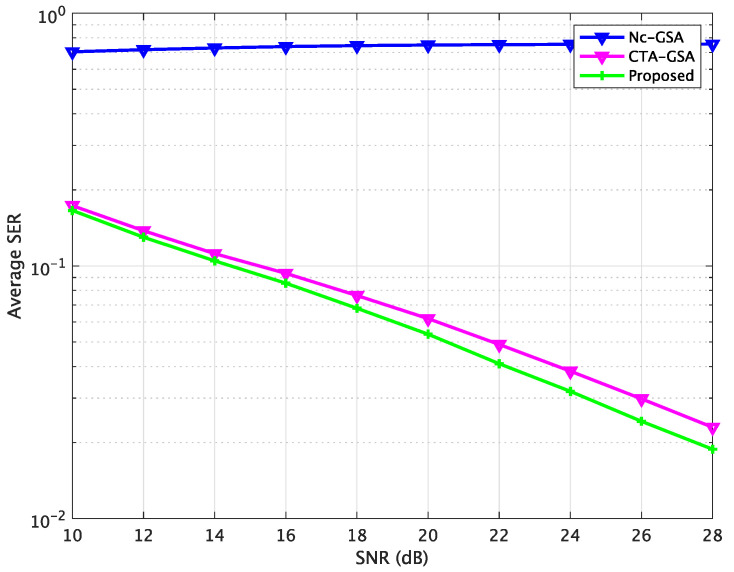
Average SER comparison between the Nc-GSA, CTA-GSA, and the proposed methods for constant channel condition of Case 2.

It can be observed from Figure 10 that blind equalization using the proposed approach achieved a smaller MSE compared to the Nc-GSA and CTA-GSA. We found that stabilized MSE levels could be obtained for 2500 data samples in the proposed method, and 4000 data samples in the CTA-GSA. The performance of average MSE for 1500 data samples was competitive with the performance of the CTA-GSA. Figure 11 illustrates the average SER for the sensor node after 6000 iterations between the Nc-GSA, CTA-GSA, and the proposed methods. Based on Figure 11, it can be observed that the proposed method outperformed the CTA-GSA by around 1 dB at the same SER $10^{-1}$. From Table 4, it can be observed that blind equalization based on the proposed methods attained improvements of almost 0.7376 and 0.0047 relative to the Nc-GSA and CTA-GSA methods, respectively, at an average SER performance.

**Figure 12:**
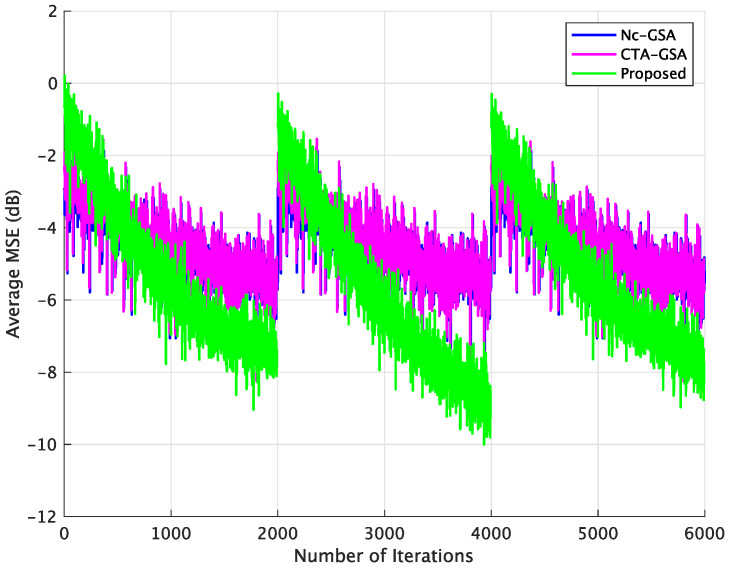
Average SER comparison between the Nc-GSA, CTA-GSA, and the proposed methods for varying channel of Case 2.

The varying channel was also used to evaluate the performance of the 20 sensor node network model. Figure 12 shows the result of the average MSE comparison between the Nc-GSA, CTA-GSA, and the proposed methods. Compared with the Nc-GSA and CTA-GSA, the average MSE level of the proposed method outperformed the average MSE level of the Nc-GSA and CTA-GSA by about 3 dB for 2000 to 4000 iterations.

## 5. Conclusions

In this paper, we propose a network topology reconfiguration approach aimed at discarding the impact of sensor nodes corresponding to ill-channels on other sensor nodes, thereby improving the performance of the blind equalizer. We utilized the generalized Sato algorithm (GSA) to design the blind equalizer. The performance of the blind equalizer was evaluated in terms of the average mean square error (average MSE) and average symbol error ratio (average SER). Subsequently, a series of numerical simulations were conducted to validate the effectiveness of the proposed method in blind equalization in comparison with the non-cooperative GSA (Nc-GSA) and the state-of-art method, the combine-then-adaptive GSA (CTA-GSA). The computation simulation results demonstrate that the performance of the blind equalizer using the proposed method exhibited a significant improvement compared to the conventional methods.

## Figures and Tables

**Table 1:** Computation complexity in *r*-th LSN.

Methods	Complex Multiplications	Complex Additions
CTA-GSA	$R_{r}^{2}D+R_{r}\left(N+3\right)$	$2R_{r}^{2}N+R_{r}\left(N+3\right)$
Proposed	${\left(R_{r}-m\right)}^{2}+\left(R_{r}-m\right)N+D\left(N+2\right)$	${\left(R_{r}-m\right)}^{2}N+\left(R_{r}-m\right)\left(N+1\right)$

“*D*” denotes all data samples, “$R_{r}$” is the total sensor number in the *r*-th LSN, and “*m*” is a neighbor of the sensor node *r*, but the weight of this sensor node is assigned to be 0.

**Table 2:** Metropolis combination weight matrix.

0.6000	0	0	0.2000	0.2000
0	0.3500	0.2500	0.2000	0.2000
0	0.2500	0.3500	0.2000	0.2000
0.2000	0.2000	0.2000	0.2000	0.2000
0.2000	0.2000	0.2000	0.2000	0.2000

**Table 3:** Average SER over WSN between the Nc-GSA, CTA-GSA, and the proposed approaches for Case 1.

	Nc-Gsa	CTA-GSA	Proposed
SER(%)	0.7547	0.0183	0.0137

**Table 4:** Average SER over WSN between the Nc-GSA, CTA-GSA, and the proposed approaches for Case 2.

	Nc-GSA	CTA-GSA	Proposed
SER(%)	0.7559	0.0230	0.0183

## Data Availability

Data are available upon request.
